# Evaluation of the NRF1-proteasome axis as a therapeutic target in breast cancer

**DOI:** 10.1038/s41598-023-43121-x

**Published:** 2023-09-22

**Authors:** Holly A. Byers, Amy N. Brooks, Janakiram R. Vangala, Jacqueline M. Grible, Alex Feygin, Charles V. Clevenger, J. Chuck Harrell, Senthil K. Radhakrishnan

**Affiliations:** Department of Pathology and Massey Comprehensive Cancer Center, Virginia Commonwealth University, Richmond, VA USA

**Keywords:** Breast cancer, Cell death, Proteasome

## Abstract

Proteasomes are multi-subunit complexes that specialize in protein degradation. Cancer cells exhibit a heightened dependence on proteasome activity, presumably to support their enhanced proliferation and other cancer-related characteristics. Here, a systematic analysis of TCGA breast cancer datasets revealed that proteasome subunit transcript levels are elevated in all intrinsic subtypes (luminal, HER2-enriched, and basal-like/triple-negative) when compared to normal breast tissue. Although these observations suggest a pan-breast cancer utility for proteasome inhibitors, our further experiments with breast cancer cell lines and patient-derived xenografts (PDX) pointed to triple-negative breast cancer (TNBC) as the most sensitive subtype to proteasome inhibition. Finally, using TNBC cells, we extended our studies to in vivo xenograft experiments. Our previous work has firmly established a cytoprotective role for the transcription factor NRF1 via its ability to upregulate proteasome genes in response to proteasome inhibition. In further support of this notion, we show here that NRF1 depletion significantly reduced tumor burden in an MDA-MB-231 TNBC xenograft mouse model treated with carfilzomib. Taken together, our results point to TNBC as a particularly vulnerable breast cancer subtype to proteasome inhibition and provide a proof-of-principle for targeting NRF1 as a viable means to increase the efficacy of proteasome inhibitors in TNBC tumors.

## Introduction

Timely degradation of misfolded, expired, or otherwise unneeded proteins is an essential aspect of cellular protein homeostasis or proteostasis^1, 2^. As the primary degradation method for the majority of intracellular proteins, the ubiquitin–proteasome system (UPS) plays an integral role in maintaining proteostasis. The UPS facilitates the ubiquitination of its substrate proteins through the concerted effort of three enzymes, E1, E2, and E3. The resultant ubiquitinated substrates are then channeled into the 26S proteasome via the 19S regulatory particle and degraded within the 20S catalytic core^1–4^. This tightly regulated system is critical for the maintenance of cellular health and survival.

The 26S proteasome is comprised of at least 33 protein subunits assembled into a highly-symmetrical, barrel-like structure that can be divided into two major sections: the 20S catalytic core and the 19S regulatory particle. The catalytic core contains two inner beta (β) rings and two outer alpha (A) rings, each comprised of seven protein subunits encoded by seven PSMB and seven PSMA genes, respectively (Supplementary Table 1)^2, 5, 6^. The 19S regulatory particle is comprised of a lid and base. The lid contains structural subunits along with ubiquitin-binding and deubiquitinating subunits encoded by at least ten PSMD genes (Supplementary Table 1)^5–7^. The base, comprised of both ATPase and non-ATPase subunits, is responsible for the unfolding and translocation of the substrate proteins into the catalytic core for proteolysis^7^.

Owing to a high degree of genetic instability, cancer cells accumulate numerous aberrations, including point mutations, deletions, and translocations in their genomes^8^. In addition, it is estimated that over 90% of solid tumors and 70% of hematologic cancers contain aneuploid cells with more than two copies of some chromosomes, resulting in a corresponding increase in the proteins being expressed from those chromosomes^9, 10^. These genetic aberrations can increase the total, mutant, and misfolded protein load in cancer cells, which in turn increases cancer cell dependence on the UPS for their degradation in order to maintain proteostasis and preserve survival^9^.

Enhanced UPS dependence in cancer cells sets the stage for use of proteasome inhibitors as cancer therapy. In addition to disrupting proteostasis, inhibition of the proteasome impacts various cellular processes controlled by the UPS that are essential for tumorigenesis and tumor progression. These include cell cycle progression, oncogenic transformation, and destruction of tumor suppressors^11–14^. To date, three proteasome inhibitors have gained Food and Drug Administration (FDA) approval: bortezomib (BTZ), carfilzomib (CFZ), and ixazomib (IXZ)^11^. Each is unique in the combination of proteasome binding mechanism, forming either an irreversible or reversible bond with the target proteasome catalytic site, and the administration route: reversible and intravenous for BTZ, irreversible and intravenous for CFZ, and reversible and oral for IXZ.

Despite predicted pan-cancer utility, evidence of in vitro efficacy, and over 1200 cancer-related clinical trials incorporating these three drugs alone or in combination with other known and potential chemotherapeutics, proteasome inhibitors are only FDA-approved for the treatment of multiple myeloma and mantle cell lymphoma^9, 11, 15, 16^. Even for these cancer types, intrinsic and acquired resistance to proteasome inhibitors is frequently observed in laboratory studies and in the clinic^17^. It is important to investigate whether combination therapies targeting resistance mechanisms have potential to enhance the chemotherapeutic effect of proteasome inhibitors and expand the repertoire of cancer types in which proteasome inhibitors are therapeutically useful to include solid tumors, such as breast cancer^17^.

One possible resistance mechanism to proteasome inhibitor therapy is the nuclear factor erythroid derived 2-related factor 1 (NRF1)-mediated proteasome bounce-back pathway. We and others have previously characterized this pathway, wherein inhibition of the proteasome leads to NRF1-dependent transcriptional upregulation of proteasome subunit (PSM) genes resulting in de novo proteasome synthesis and a rescue of proteasome activity^18, 19^. NRF1 is cotranslationally inserted into the endoplasmic reticulum (ER) membrane via a Sec61-dependent pathway and glycosylated, then extracted into the cytosol via the action of the ATPase p97/VCP and deglycosylated by the p97-interacting glycanase NGLY1^18–21^.

When the proteasome is active, NRF1 is maintained at low basal levels in the cell by constant 26S proteasome-mediated degradation, completing the ER-associated protein degradation (ERAD) cycle^18, 19, 22^. When proteasome activity is inhibited or impaired, NRF1 is cleaved to its active form by the protease DDI2 in the cytosol and translocated into the nucleus to bind co-factors, such as MafG and TIP60, where it functions as an active transcription factor^18–20, 23–26^. The NRF1 activation pathway culminates in increased expression of the PSM genes to initiate the proteasome bounce-back response that can attenuate proteasome inhibitor-induced cell death^18, 19^.

Reversible proteasome inhibition allows cells to recover proteasome activity via the NRF1-mediated proteasome bounce-back response together with a simple dissociation of the inhibitors from the proteasomes. On the other hand, the bounce-back response is the only option for overcoming irreversible proteasome inhibition^18^. Rapid clearance of the irreversible proteasome inhibitor carfilzomib from patient blood, occurring within as much as an hour of administration, prevents inhibition of the newly-assembled proteasomes resulting from the bounce-back response, thus severely limiting the duration of proteasome inhibition that is attainable^18, 27–29^. Inhibiting the bounce-back response by depletion of NRF1 was shown to sustain the duration of proteasome inhibition, thus potentiating irreversible proteasome inhibitor-induced cell death, in triple negative breast cancer (TNBC) and osteosarcoma cell lines in vitro^18^.

To date, the in vivo efficacy of targeting NRF1 to inhibit the bounce-back response in combination with irreversible proteasome inhibition has not been investigated. In this study, we found that although expression of PSM genes is elevated in all subtypes of breast cancer, the basal-like/TNBC subtype is the most sensitive to proteasome inhibitors. Furthermore, we show that depletion of NRF1 sensitizes tumors to irreversible proteasome inhibition in a TNBC xenograft mouse model.

## Results

### Increased 26S proteasome gene expression is associated with a worse breast cancer prognosis

**Figure 1:**
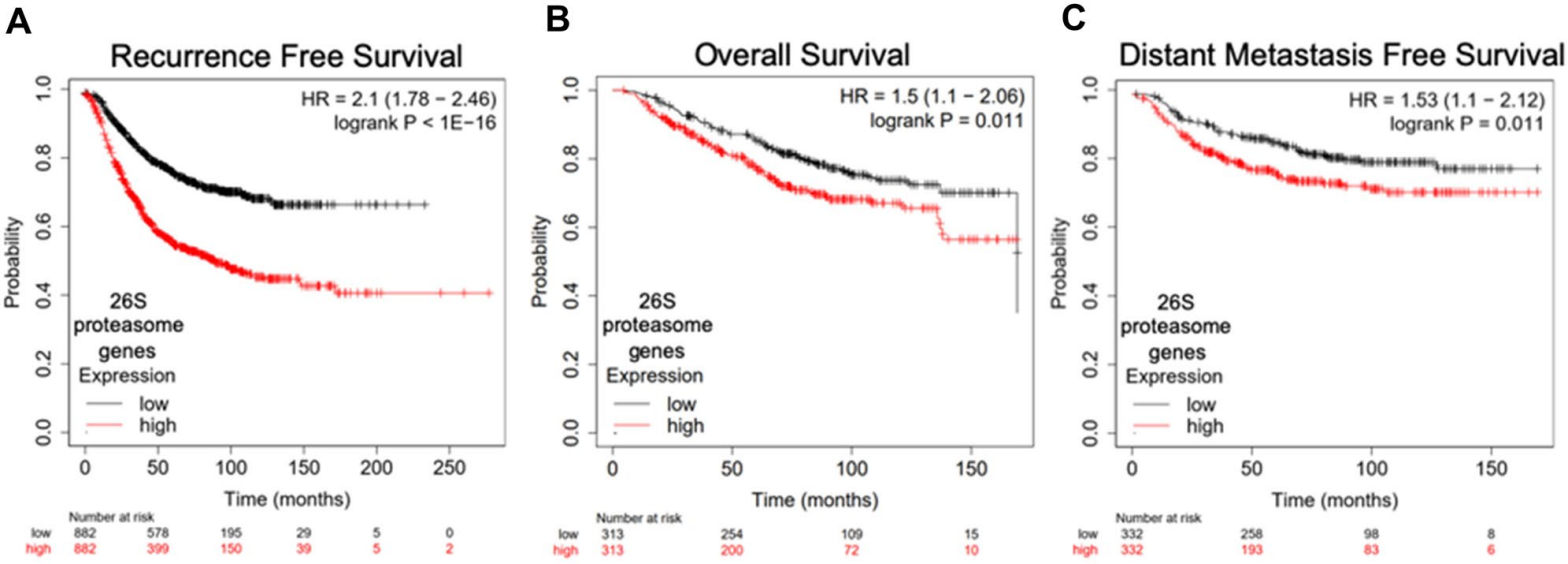
Increased expression of proteasome subunit genes is associated with a worse breast cancer prognosis. High expression of the 26S proteasome gene signature in primary tumor tissue is associated with decreased (**A**) recurrence free survival (n = 882), (**B**) overall survival (n = 313), and (**C**) distant metastasis free survival (n = 332) in patients with breast cancer. Data and graphs were generated by the breast cancer mRNA gene chip dataset in the KM Plotter database using median gene signature expression to divide patient samples into high and low expression.

Increased proteasome activity has been observed across human cancers, correlating with cancer-related phenotypes, including enhanced proliferation and pro-inflammatory NF-κB pathway activity^30, 31^. More specifically in breast cancer, a study with matched tumor and control tissues from the Cancer Institute of New Jersey Tissue Retrieval Service showed that > 90% of the breast cancer samples (n = 25) showed activation of the UPS, with higher proteasome activity correlating with increased levels of proteasome subunits^32^. Here, we asked if proteasome gene expression levels could be linked to patient outcomes in breast cancer. To this end, we analyzed the 26S proteasome subunit gene signature (Supplementary Table 1) in breast cancer using the Kaplan–Meier plotter (KM-Plotter) database^33, 34^. Our analysis revealed that high 26S subunit gene expression is associated with significantly decreased recurrence free survival, overall survival, and distant metastasis free survival (Fig. 1A–C). As a comparison, we analyzed a gene signature consisting of select target genes of tumor suppressor p53 (Supplementary Table 1) in KM-plotter. We found that increased expression of this signature is associated with a better prognosis in terms of recurrence-free and overall survival, but has no correlation with distant metastasis-free survival (Supplementary Fig. 1A-C).

### Increased proteasome gene expression in breast cancer is maintained across subtypes

**Figure 2:**
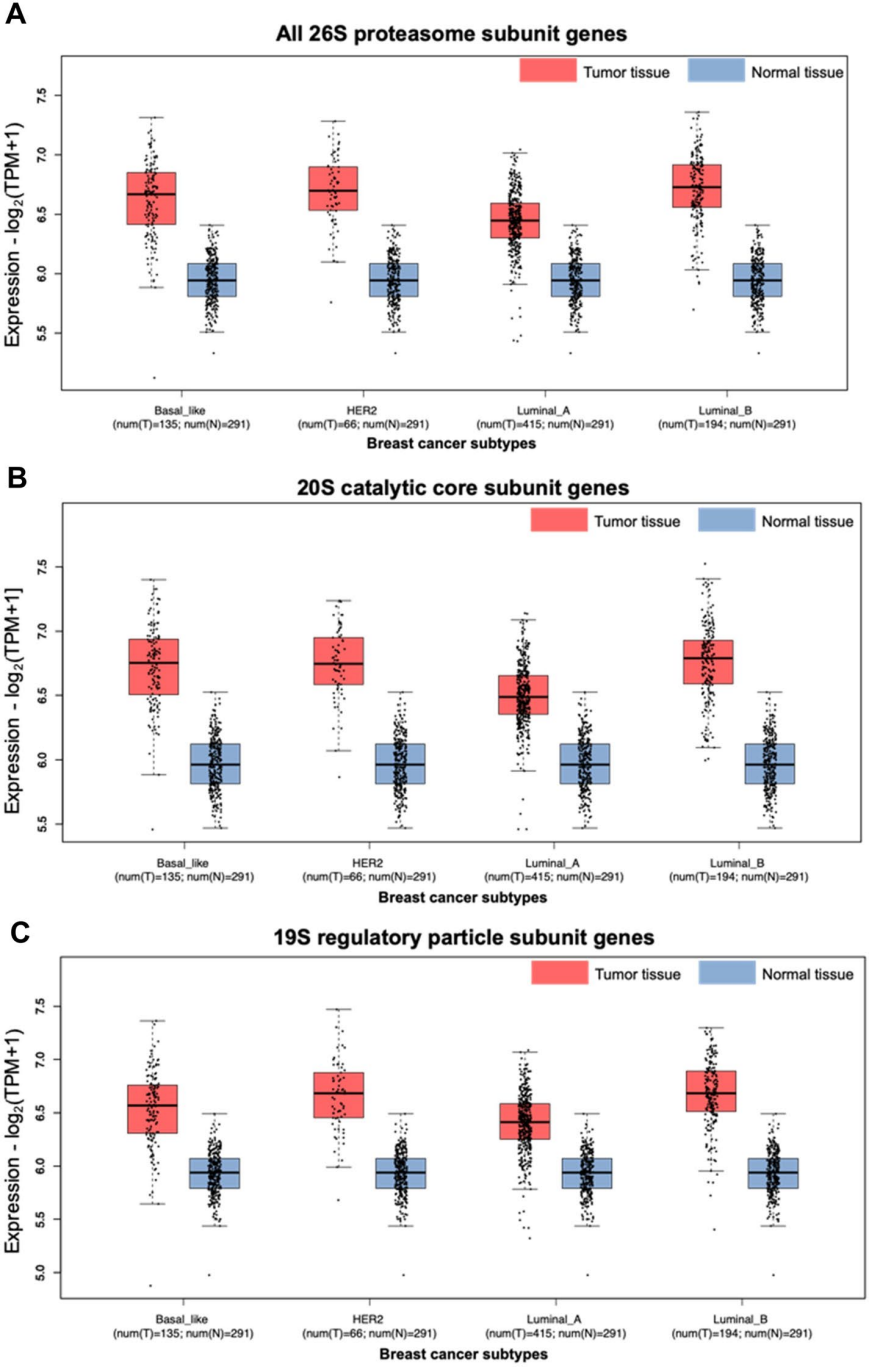
Proteasome subunit gene signatures show increased expression across breast cancer subtypes when compared to normal breast tissue. Expression of the 26S proteasome (**A**), 20S catalytic core (**B**), and 19S regulatory subunit (**C**) gene signatures are increased in primary breast cancer tumor tissue compared to normal breast tissue (n = 291) across breast cancer subtypes. Data and graphs were generated by the GEPIA2 database. Each point represents a patient sample’s average expression for the gene signature; basal-like (n = 135), HER2-enriched (n = 66), luminal A (n = 415), and luminal B (n = 194).

Next, we asked if the high levels of proteasome gene expression observed in breast cancer is a more general phenomenon or could be restricted to certain subtypes and/or associated with other cancer cell characteristics. Analysis of the 26S proteasome subunit gene signature using the GEPIA2 database^35^ showed a consistent increase in its expression in primary breast tumor tissue compared to normal tissue across all of the breast cancer subtypes: basal-like TNBC, HER2-enriched, luminal A, and luminal B (Fig. 2A). In contrast, a p53 target gene signature showed a decrease in basal-like and HER2-enriched, but not luminal, subtypes (Supplementary Fig. 1D).

In some instances, the 20S catalytic core can function without the 19S regulatory particle to degrade proteins in an ubiquitin-independent fashion^36–38^. Without the 19S ATPase subunits to facilitate protein unfolding, substrates of the ubiquitin-independent degradation pathway must already be partially unfolded. Thus, proteins with unstructured regions due to mutations or oxidative damage are typical substrates of this 20S proteasome pathway^37, 39, 40^. As misfolded protein accumulation and oxidation are both common occurrences in cancer cells, we asked if there is a selective increase in 20S expression resulting in an obvious shift towards ubiquitin-independent protein degradation. However, the primary breast cancer tumor tissues across all subtypes showed similar increases in 20S and 19S proteasome subunit gene expression (Fig. 2B,C). This indicates that the ubiquitin-dependent 26S proteasome pathway likely remains active and contributes to the observed increase in proteasome activity in breast cancer tissue across subtypes.

### Triple-negative breast cancer (TNBC) cells are more sensitive to proteasome inhibition

**Figure 3:**
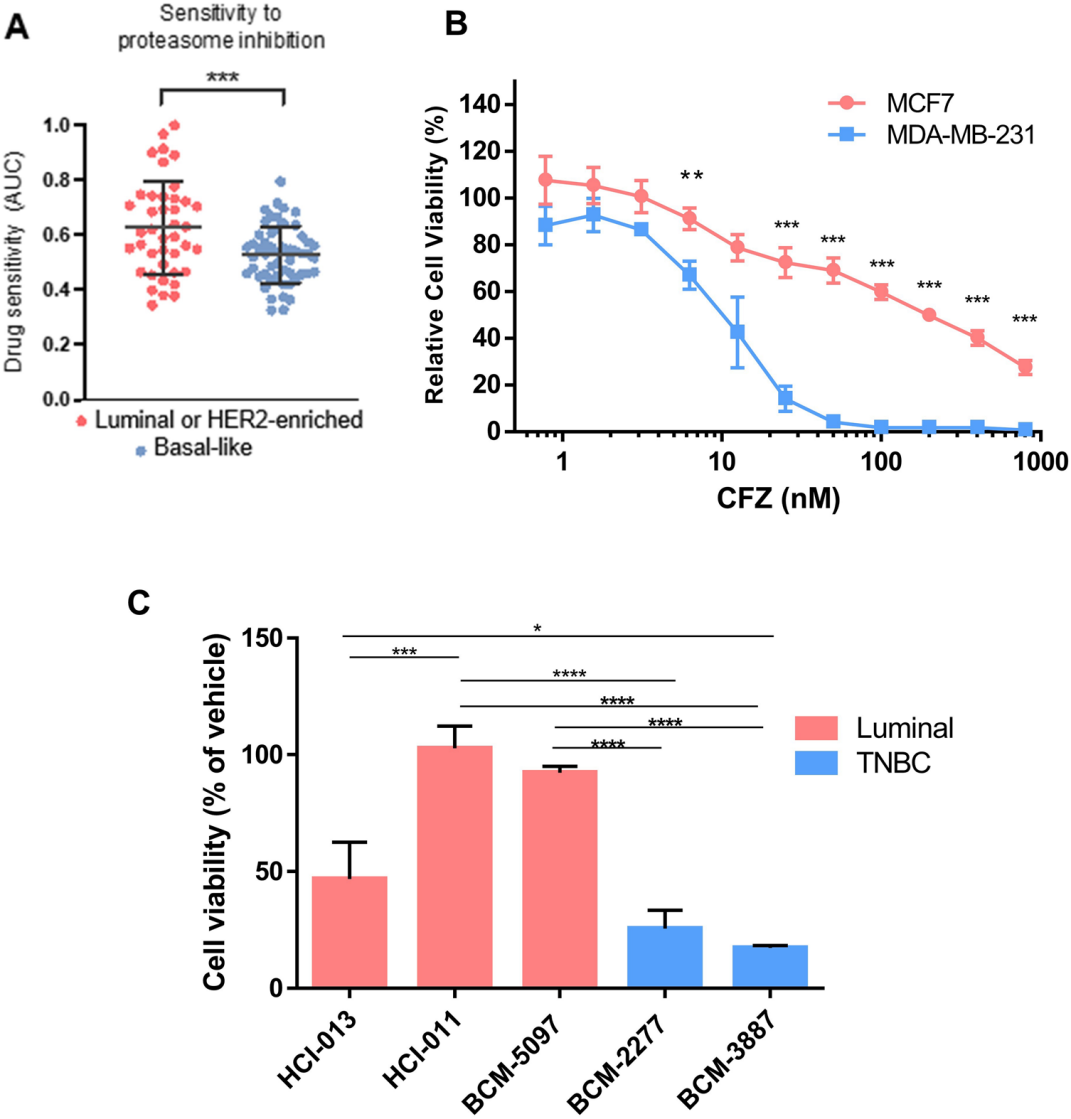
Triple negative breast cancer PDX and cell line models are more sensitive to proteasome inhibitor treatment compared to luminal breast cancer in vitro. (**A**) Sensitivity scores (AUC) to the proteasome inhibitors bortezomib, carfilzomib, ixazomib, delanzomib, and oprozomib in luminal (ER+) and HER2-enriched cell lines (n = 41) compared to basal-like TNBC cell lines (n = 53). Unpaired student’s *t* test; p value = 0.0008. Data generated using DepMap. (**B**) Luminal breast cancer-derived MCF7 control (EGFP sgRNA expressing) and triple negative breast cancer-derived MDA-MB-231 control cells (EGFP sgRNA expressing) were treated with increasing doses of CFZ (0.7825–800 nM) for 48 h and analyzed by a luminescent cell viability assay (CellTiter-Glo). Relative cell viability was determined via comparison to viability of vehicle/DMSO-treated cells (set at 100% viability). Statistical significance was determined using the Holm-Sidak method, with alpha = 5.000%. Error bars indicate standard deviation (n = 3); (**denotes p ≤ 0.005; ***denotes p < 0.0005). (**C**) PDX tumor cell suspension culture viability (CellTiter-Glo) after 72 h of treatment with 10 nM carfilzomib. Percent viability was normalized to vehicle-treated controls. Statistical significance was determined using One-way ANOVA (p < 0.0001) and Tukey’s multiple comparisons test. Error bars represent standard deviation between independent experiments (n = 3); *denotes p < 0.05, ***denotes p < 0.0001, and ****denotes p < 0.00001.

Given that PSM gene expression is elevated in all breast cancer subtypes, we asked if this could result in a pan-breast cancer enhanced susceptibility to proteasome inhibitors. To contrast these findings in a larger-scale dataset, we utlilized the Cancer Dependency Map Project (DepMap), a database that includes genetic vulnerabilities and drug sensitivity metrics for numerous cancer cell lines^41, 42^. Focusing on the breast cancer cell lines from the DepMap collection, we analyzed their sensitivity to the proteasome inhibitors bortezomib, carfilzomib, ixazomib, delanzomib, and oprozomib (Supplementary Table 2). Interestingly, we found that basal-like TNBC cell lines were significantly more sensitive to the proteasome inhibitors than the luminal and HER2-enriched subtype cell lines (Fig. 3A). We then tested whether TNBC breast cancer cells (MDA-MB-231) were more sensitive to carfilzomib treatment in vitro compared to luminal, ER + breast cancer cells (MCF7). We treated cells with serial dilutions (0.7825–800 nM) of FDA-approved proteasome inhibitor carfilzomib (CFZ) for 48 h and performed luminescent ATP-based cell viability assays (CellTiter-Glo, Promega). We observed TNBC MDA-MB-231 cells were significantly more sensitive to CFZ treatment and had increased cell death at lower doses of the drug when compared to luminal estrogen receptor (ERα) and progesterone receptor (PR) positive MCF7 cells (Fig. 3B). Viability was reduced to near 0% for both MDA-MB-231 at doses of CFZ exceeding 50 nM, whereas MCF7 cells retained some viability at higher doses of carfilzomib treatment (50–800 nM). To further compare breast cancer cell response to carfilzomib, we utilized breast cancer patient-derived xenograft (PDX) models of various subtypes and treated them with 10 nM carfilzomib in basal-like TNBC (BCM-2277, BCM-3887) and luminal ERα+, PR+ positive PDX lines (HCI-011, HCI-013, and BCM-5097). We recapitulated in these experiments that TNBC cells were significantly more sensitive to proteasome inhibition when compared to ERα-positive PDXs (Fig. 3C).

### NRF1 depletion sensitizes MDA-MB-231 xenografts to irreversible proteasome inhibition

**Figure 4:**
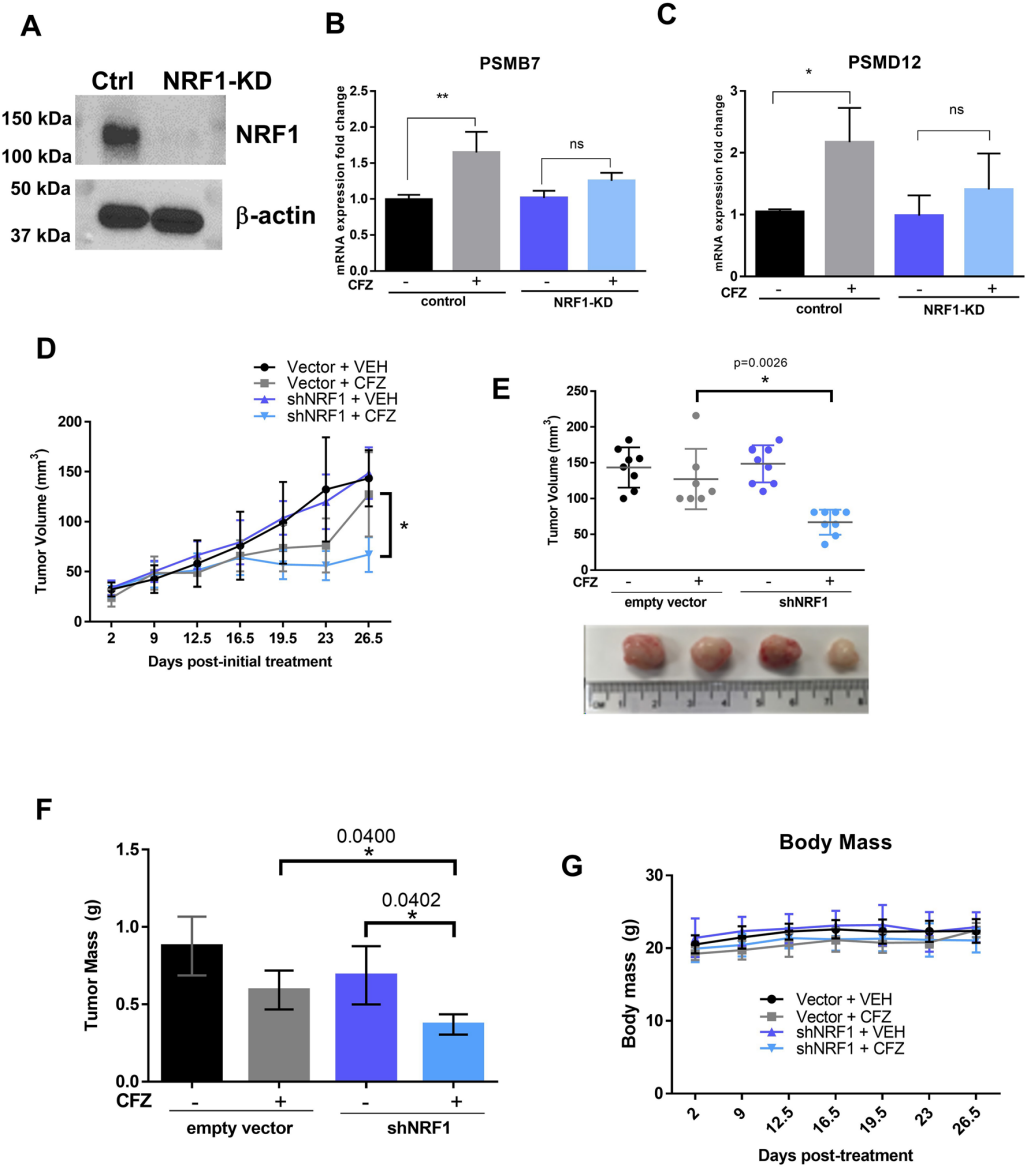
NRF1-knockdown in a TNBC xenograft mouse model sensitizes tumors to proteasome inhibition. (**A**) MDA-MB-231 control and shNRF1 cells were treated with carfilzomib (200 nM) for 4 h, where NRF1 level was analyzed by Western blot to confirm shNRF1 knockdown. β-actin was used as a loading control. The original blots are displayed in supplementary materials. (**B**,**C**) qRT-PCR results for PSM genes (PSMB7 and PSMD12, n = 3) in luminal breast cancer subtype MDA-MB-231 cells treated with DMSO or CFZ for 16 h. Statistical significance was determined using One way ANOVA with Tukey’s multiple comparisons test (significant p value < 0.05; **denotes p-value < 0.01 and *denotes p =  < 0.05, “ns” denotes not significant. (**D**,**E**) MDA-MB-231 empty vector control or shNRF1 cells were seeded to NOD-SCID mice and allowed to form palpable tumors, followed by treatment with vehicle or CFZ (5 mg/kg) for 4 weeks (n = 8 per group). (**D**) Tumor volume was measured with manual calipers at the time points indicated and plotted over time, and (**E**) plotted as final volume, with images of representative tumors. The modified ellipsoid formula was used to determine tumor volume (mm^3^). Unpaired student’s *t* test; p value = 0.0026. (**F**) Tumors were dissected after the final volume measurement and weighed to determine tumor mass (g). Unpaired student’s *t* test; p value = 0.0400 and p = 0.0402 between indicated groups. (**G**) Mice were weighed at the time points indicated. Error bars denote standard deviation between measurements for each mouse in the indicated cell line and treatment cohorts.

Although PSM gene expression is elevated in all subtypes of breast cancer, our observation that TNBC is particularly susceptible to proteasome inhibitors in vitro prompted us to test this subtype further in an in vivo setting. Unlike cultured cells, which can be continuously incubated with proteasome inhibitors, the duration of chemical proteasome inhibition that can be achieved in patients or animal models is limited by rapid clearance of these drugs from the blood. Although an irreversible proteasome inhibitor is expected to block activity of the pre-existing proteasomes in tumors, these cells could still recover their proteasome activity by invoking the NRF1-mediated bounce-back pathway to produce de novo, uninhibited proteasomes. We hypothesized that the depletion of NRF1 in both secondary breast cancer cell lines grown in vitro and xenograft tumor cells would overcome this potential resistance mechanism and confer sensitivity to the irreversible proteasome inhibitor carfilzomib, despite its expected rapid clearance from the blood. To test this hypothesis, we utilized TNBC (ERα-/PR-/HER2-) MDA-MB-231 cells constitutively expressing either empty vector control or an shRNA targeting NRF1 (MDA-MB-231-shNRF1)^18^. Using immunoblots, we first treated both cell lines with CFZ to visualize NRF1 accumulation, and confirmed that there is a significant level of knockdown of NRF1 in these cells when compared to control (Fig. 4A). We also confirmed via qRT-PCR that proteasome subunit genes, such as PSMB7 and PSMD12, which are induced by NRF1 when the proteasome is inhibited, had attenuated expression in the NRF1-knockdown breast cancer cell lines (Fig. 4B,C).

Next, we induced xenograft mammary tumors in female NOD-SCID- IL2Rγ (NSG) mice using MDA-MB-231-shNRF1 or control cell lines. The animals received vehicle or carfilzomib, the only FDA-approved irreversible proteasome inhibitor, twice weekly intravenously. The carfilzomib dosing schedule and delivery method was chosen to mirror its clinical use for FDA-approved indications of relapsed or refractory multiple myeloma^43^. We found that carfilzomib treatment of NRF1-depleted tumors resulted in significantly reduced tumor growth, along with a corresponding decrease in final tumor volume and tumor mass (Fig. 4D–F). Of note, the animals did not display any signs of drug toxicity throughout our treatment period and did not exhibit any appreciable weight loss (Fig. 4G). Overall, our results provide proof-of-principle for targeting the NRF1 pathway to sensitize TNBC tumors to irreversible proteasome inhibitor therapy.

## Discussion

Increased proteasome activity has been observed across multiple human cancer types, including breast cancer^30–32^. However, previous studies have indicated somewhat conflicting breast cancer subtype-specific vulnerability to proteasome inhibitors in vitro^16, 44–50^. We utilized patient tissue-derived gene expression databases to demonstrate that increased PSM gene expression for the total 26S proteasome, including the 19S regulatory particle and 20S catalytic core, is conserved across breast cancer subtypes. Despite this widespread increase in proteasome gene expression, our experiments with clinically relevant breast cancer PDXs indicate that the TNBC subtype is substantially more sensitive to proteasome inhibitors. This is consistent with some earlier reports that suggested an “addiction” to proteasome activity in established TNBC cell lines^48, 51^. The exact molecular mechanism behind this phenomenon remains to be elucidated.

Although experimental models have extensively predicted pan-cancer utility of proteasome inhibitors, its use as a monotherapy has failed to show significant clinical activity in any type of solid tumor, including breast cancer^16, 44–50, 52^. Even in multiple myeloma, for which bortezomib is FDA-approved as a first line therapy, the drug fails to induce major clinical responses in more than half of patients or fully cure any patient^53, 54^. Combination therapies targeting resistance mechanisms to potentiate proteasome inhibition could improve the clinical efficacy of proteasome inhibitors in their current approved usage settings, and even expand the repertoire of cancer types that can be targeted with the use of proteasome inhibitors^17^. Future studies are needed to determine whether this therapeutic strategy could improve patient outcomes.

A potential route by which cancer cells can overcome proteasome inhibition is by invoking the NRF1-mediated bounce-back response to transcriptionally upregulate PSM genes, resulting in de novo proteasome synthesis and a subsequent rescue of proteasome activity^18, 19^. Our current experimental data builds on an earlier observation that depletion of NRF1 sensitized TNBC cells to irreversible proteasome inhibition in vitro^18^. Though other methods of inhibiting the proteasome bounce-back response pathway have been evaluated by using inhibitors of NGLY1 and p97^21, 55, 56^, none have directly targeted the critical transcription factor NRF1 to potentiate the action of irreversible proteasome inhibition in vivo. Our results from this study demonstrate that depleting NRF1 to inhibit the proteasome bounce-back bounce sensitized TNBC xenograft tumors to carfilzomib treatment in mice.

Although we focused on a proteasome-sensitive TNBC model in our current work, it is possible that this general strategy of targeting the NRF1-proteasome axis could be successful in other breast cancer subtypes or even in other types of solid tumors. Thus, further research into this area could expand the reach of proteasome inhibitors to treat cancers with limited therapeutic options.

## Methods

### Kaplan–Meier (KM) plotter database analysis

Gene signatures for the 26S proteasome (all genes from the 19S regulatory particle and 20S catalytic core gene signatures) and p53 target genes were analyzed using the KM plotter mRNA gene chip database for breast cancer (kmplot.com/analysis/)^5, 6, 33, 57^. The gene signatures and probe IDs used for the analysis are compiled in Supplementary Table 1. KM plots for the tumor suppressor p53 target gene signature were generated as a control (Supplementary Fig. 1). KM plots for recurrence free survival, overall survival, and distant metastasis free survival were generated using the median expression to divide patients into “high” and “low” expression for each gene signature. There were no analysis restrictions used for breast cancer subtypes or cohorts.

### GEPIA2 database analysis

Differential expression between breast cancer and normal tissue was evaluated using the “Expression DIY” tool housed under the “Expression Analysis” function in GEPIA2 (gepia2.cancer-pku.cn)^35^. The Box Plot pane was used to analyze gene signatures for the 19S regulatory particle, 20S catalytic core, 26S proteasome (all genes from the 19S regulatory particle and 20S catalytic core gene signatures), and p53 target genes (Supplementary Table 1)^5, 6, 57^. Expression analysis for the tumor suppressor p53 target gene signature was generated as a control (Supplementary Fig. 1D). The prepopulated settings were used for “|Log_2_FC| Cutoff” (1), “p-value Cutoff” (0.01), “Log Scale” (yes; log_2_(TPM + 1)), “Jitter Size” (0.4), and “Matched Normal data” (Match TCGA normal and GTEx data). We used the subtype filter for breast cancer (BRCA), selecting all subtypes (Basal-like/Triple negative, HER2 + non-luminal, Luminal A, and Luminal B) to be graphed in a separated plot type.

### DepMap portal analysis

The “Data Explorer” tool in DepMap (depmap.org/portal/) was used to determine proteasome inhibitor sensitivity in breast cancer cell lines in vitro. Cell lines were filtered by “Breast.” Sensitivity AUC values were extracted from carfilzomib (BRDBRD-K15179879-001-03-2), ixazomib (BRDBRD-K78659596-001-03-9), oprozomib (BRDBRD-A36331462-001-02-1), delanzomib (BRDBRD-K59325863-001-02-8), and bortezomib (BRDBRD-K88510285-001-17-8) AUC Drug sensitivity AUC (PRISM Repurposing Secondary Screen) 1811 Public Tentative downloaded datasets.

### Breast cancer PDX models and cell viability assays

Breast cancer PDX model acquisition, preparation of tumor cell suspensions, drug treatments, and cell viability assays were described by Turner et al.^58^. Briefly, luciferase-expressing PDX cell suspensions were plated in 96-well plates at 25,000 cells per well and treated with 10 nM proteasome inhibitor carfilzomib at the indicated concentrations for 72 h. After incubation, the CellTiter-Glo assay (Promega) was performed according to standard protocol and luminescence was quantified using a POLARStar Optima cell microplate reader. Luminescence was normalized to vehicle-treated control cells.

### Cell lines and culture conditions

The MDA-MB-231-shNRF1 and corresponding control PRS-puromycin vector cell lines were previously described by Radhakrishnan et al.^18^. The MDA-MB-231 DDI2^−/−^ cell line was previously described by Northrop et al.^26^. All secondary cell lines (MDA-MB-231-derived and MCF7) were grown in Dulbecco’s modified Eagle’s medium (DMEM) supplemented with 10% fetal bovine serum (Atlanta Biologicals) and penicillin and streptomycin (Invitrogen) at 37 °C in a humidified incubator with 5% CO_2_.

### Western blot analysis

Cell lysate preparation and western blot analysis was done as previously described^26^. Briefly, cells were collected by scraping, washed with cold PBS, and pelleted by centrifugation. Pellets were resuspended in RIPA lysis buffer (50 mM Tris–HCl pH 8.0, 150 mM NaCl, 0.1% Triton X-100, 0.5% Sodium deoxycholate, 0.1% SDS) supplemented with protease and phosphatase inhibitor cocktail (Thermo Fisher Pierce, Waltham, MA, USA), incubated on ice for 30 min, then centrifuged at 14,000 rpm for 20 min at 4 °C. Protein was quantified using the Pierce BCA Protein Assay Kit (Thermo Scientific, Waltham, MA, USA). Sample concentrations were equalized and Laemmli sample buffer (Bio-Rad, Hercules, CA, USA) was added to 1×.

For western blot analysis, lysate samples were boiled for approximately 7 min, then separated by SDS-PAGE on 10–15% acrylamide gels in SDS-PAGE buffer (250 mM tris, 1.92 M glycine, 1% SDS). Electrophoresed proteins were transferred to polyvinylidene difluoride membranes using the Trans-Blot Turbo Transfer System (Bio-Rad, Hercules, CA, USA) in transfer buffer (48 mM Tris, 39 mM glycine, 20% methanol) and blocked for 1 h with 5% non-fat dry milk powder in tris-buffered saline with tween (TBST; 50 μM Tris-Base, 150 μM NaCl, 0.001% Tween 20). Membranes incubated with primary antibodies overnight at 4 °C, washed three times with TBST, incubated with secondary antibody at room temperature for 1 h, and washed again before incubation with Pierce ECL Western Blotting Substrate (Thermo Scientific, Waltham, MA, USA) and imaged using LI-COR or BioRad imager. The antibodies used were specific for NRF1 (1:1000, Cell Signaling Technology, Danvers, MA, USA) and β-actin (1:10,000, Millipore Sigma, Burlington, MA, USA). The secondary antibodies used were rabbit IgG HRP and mouse IgG HRP (1:10,000; both from Bio-Rad, Hercules, CA, USA).

### qRT-PCR for PSM genes in breast cancer lines

RNeasy kit with DNase treatment (Qiagen, Germantown, MD, USA) was used to isolate RNA from flash frozen cell pellets. The iScript cDNA synthesis kit (Bio-Rad, Hercules, CA, USA) was used to convert 1 µg of RNA to cDNA. Quantitative reverse PCR (qPCR) was performed with iTaq universal SYBR green supermix (Bio-Rad, Hercules, CA, USA) in the C1000 Touch Thermal cycler (Bio-Rad, Hercules, CA, USA). Data was analyzed using CFX manager 3.1 (BioRad, Hercules, CA, USA). Levels of GAPDH expression were used for normalization. Statistics were determined by Student’s unpaired t-test. The forward and the reverse primers used for the qPCR reactions are as follows: PSMB7 (5′-TGC AAA GAG GGG ATA CAA GC-3′ and 5′-GCA ACA ACC ATC CCT TCA GT-3′), PSMD12 (5′-GTG CGC GAC TGA CTA AAA CA-3′ and 5′-TAG GCA GAG CCT CAT TTG CT-3′), and GAPDH (5′-AAC TTT GGC ATT GTG GAA GG-3′ and 5′-GGA TGC AGG GAT GAT GTT CT-3′).

### CellTiter-Glo viability assays in breast cancer lines

Breast cancer cell lines were plated at 12,000 cells/well, which put them at approximately 70% confluency in 96-well plates. Cells were allowed to adhere to plates overnight, and then treated for 48 h in triplicate at serial dilutions of carfilzomib (CFZ) with concentrations from 0.78125 nM to 800 nM. Treated cells were placed in a humidified incubator (37C, 5% CO_2_). After incubation, the CellTiter-Glo assay (Promega) was performed according to standard protocol and luminescence was quantified using a POLARStar Optima cell microplate reader.

### Mouse xenograft model

All animal studies were performed with approval from the Virginia Commonwealth University (VCU) Institutional Animal Care and Use Committee (IACUC) (Protocol AD10001417) in accordance with IACUC guidelines and regulations. The results of experiments involving animals are reported in compliance with the ARRIVE guidelines 2.0. Female NOD-SCID-IL2Rγ (NSG) mice aged 4–6 weeks were subcutaneously injected unilaterally into the right distal mammary fat pad, proximate to the 5th teat, with 500,000 MDA-MB-231 shNRF1 or vector control cells (n = 20 mice per cell line) resuspended in Matrigel. The total injection volume was 100 µL. At 11 days post-injection, we began twice weekly treatment of the mice with carfilzomib (5 mg/kg) or equal volume Captisol by tail vein injection for 4 weeks. Tumor size was measured by manual calipers and tumor volume was generated using the modified ellipsoid formula^59^.

### Supplementary Information

Supplementary Information 1.

Supplementary Information 2.

## Data Availability

The following online databases/tools were used—Kaplan Meier plotter (kmplot.com/analysis/), GEPIA2 database (gepia2.cancer-pku.com), and DepMap portal (depmap.org/portal/). The datasets used and/or analysed during the current study are available from the corresponding author on reasonable request.
