# Supplementary figures and images for: Evaluation of the NRF1-proteasome axis as a therapeutic target in breast cancer
Source: Sci Rep. 2023 Sep 22;13:15843. doi: 10.1038/s41598-023-43121-x (PMC10516926; doi:10.1038/s41598-023-43121-x)

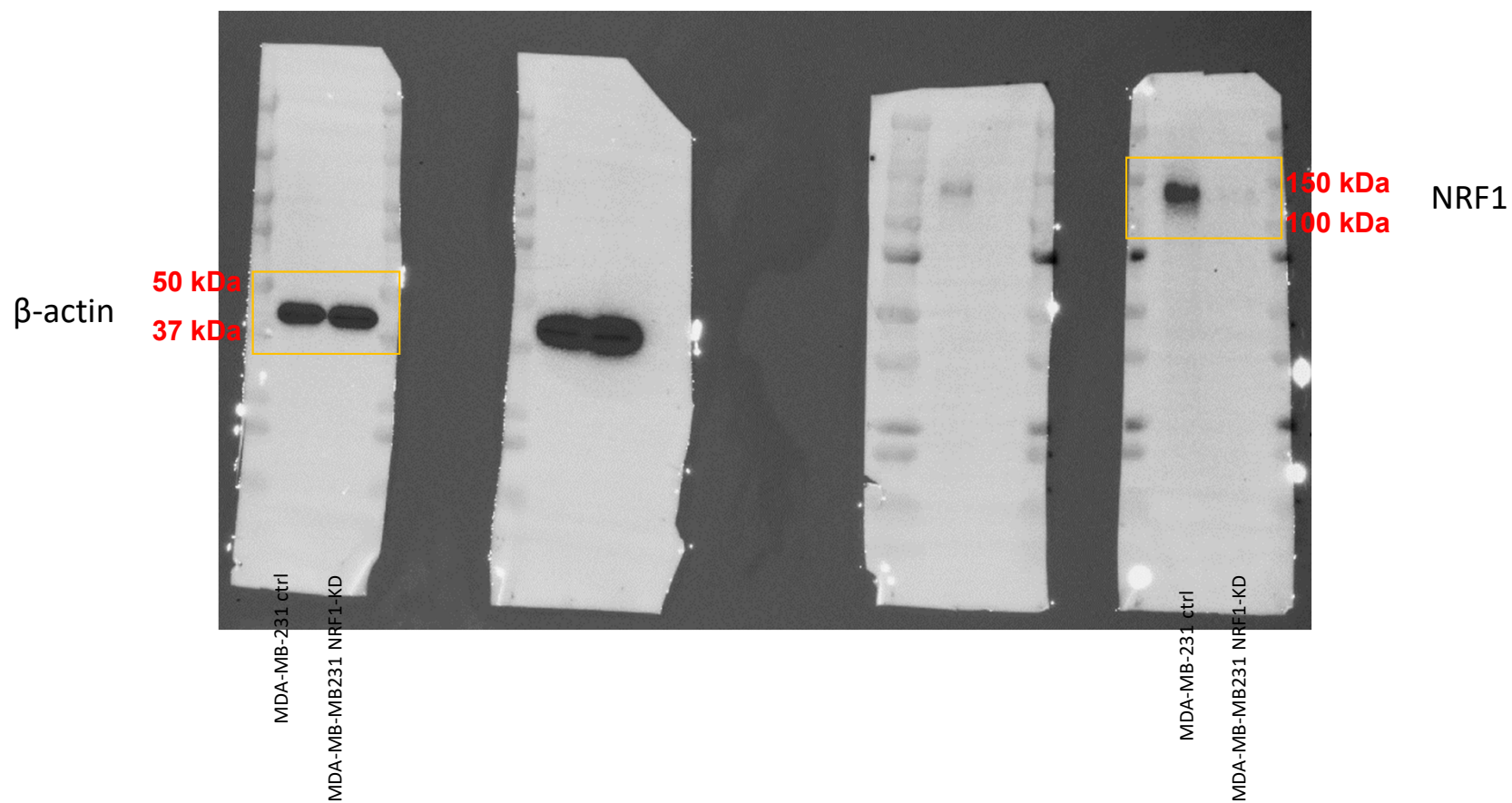

Uncropped membranes for the panels in Figure 4A.

Supplement: Supplementary file 1 — Supplementary Information 1. [file 41598_2023_43121_MOESM1_ESM.pdf]
