# Supplementary material for: Evaluation of the NRF1-proteasome axis as a therapeutic target in breast cancer
Source: Sci Rep. 2023 Sep 22;13:15843. doi: 10.1038/s41598-023-43121-x (PMC10516926; doi:10.1038/s41598-023-43121-x)
Supplement: Supplementary file 2 — Supplementary Information 2. [file 41598_2023_43121_MOESM2_ESM.docx]

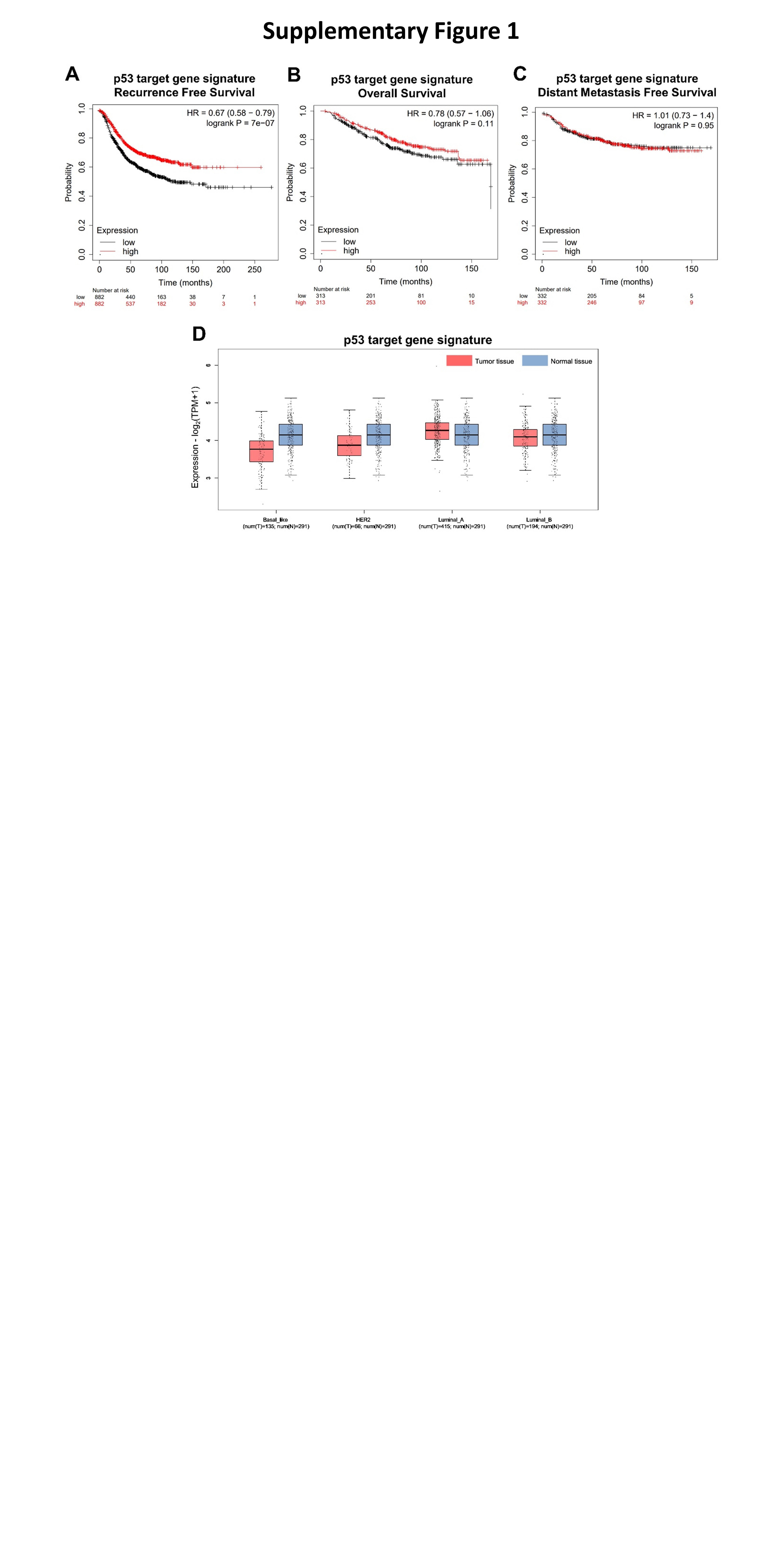


**Supplementary Figure 1.** **Expression of a p53 target gene signature in breast cancer.** (**A-C**) High expression of the p53 target gene signature in primary tumor tissue is associated with increased recurrence free survival (n=882) and overall survival (n=313), and does not predict distant metastasis free survival (n=332) in patients with breast cancer. Data and graphs were generated by the breast cancer mRNA gene chip dataset in the KM Plotter database using median gene signature expression to divide patient samples into high and low expression. (**D**) Expression of the p53 target gene signature is decreased or unchanged in primary breast cancer tumor tissue compared to normal breast tissue (n=291) across breast cancer subtypes; basal-like (n=135), HER2-enriched (n=66), luminal A (n=415), and luminal B (n=194). Data and graphs were generated by the GEPIA2 database. Each point represents a patient sample’s average expression for the gene signature.

**Supplementary Table 1.** Gene names and probe IDs used in the KM plotter database to generate the KM plots in Figure 1B-D (26S proteasome gene signature) and Appendix B (p53 target gene signature). The 26S proteasome gene signatures incorporates all genes and probe IDs listed in the 19S regulatory particle and 20S catalytic core gene signatures.


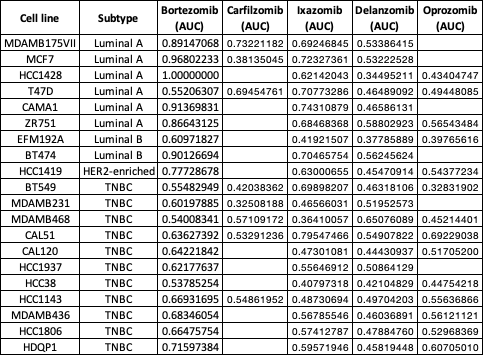


**Supplementary Table 2.** Cell lines, subtypes, and AUC sensitivity scores for the proteasome inhibitors indicated from the DepMap database used to generate Figure 3A.
